# Anatomy of Internal Iliac Artery and Its Ligation to Control Pelvic Hemorrhage

**DOI:** 10.31729/jnma.4958

**Published:** 2020-10-31

**Authors:** Ramesh Shrestha, Sapana Shrestha, Sarita Sitaula, Pritha Basnet

**Affiliations:** 1 Department of Obstetrics and Gynaecology, B.P. Koiraia Institute of Health Sciences, Dharan, Nepal; 2 Master in Nursing, Women's Health and Development, Maharajgunj Nursing Campus, Kathmandu, Nepal

**Keywords:** *internal iliac artery ligation*, *operative complications*, *pelvic anastomoses*, *pelvic hemorrhage*, *retroperitoneal anatomy*

## Abstract

Pelvic hemorrhage is a major cause of maternal morbidity and mortality in developing countries. A sound clinical judgment, adequate assessment, and preparation of the patient are the best preoperative means to avoid its occurrence. Bilateral internal iliac artery ligation is a life-saving procedure to control massive obstetric and gynecological hemorrhage when other measures fail. This procedure significantly reduces the pulse pressure and rate of blood flow abolishing the'trip-hammer effect' of arterial pulsation and subsequently resulting in sluggish blood flow allowing effective thrombosis within the small bleeding vessels. This has helped to save many lives and uteruses for more than a century. No tissue necrosis occurs due to ample collateral circulation in the pelvis from the major pelvic anastomoses. An increased understanding of retroperitoneal anatomy and regional variations of the internal iliac artery is needed to reduce the risk of intraoperative and postoperative complications.

## INTRODUCTION

The historical background of ligating the internal iliac artery (IIA) also called a hypogastric artery to control pelvic hemorrhage in gynecological surgery is not clear. The IIA ligation can be traced back to 1812 when it was done unilaterally for a gluteal aneurysm. Howard Kelly^1^ of Baltimore in 1894 was the first to ligate both IIA along with ovarian arteries for bleeding cervical carcinoma with extensive broad ligament involvement. The procedure was extensively investigated by Burchell RC^2^ in 1968 but later introduced by Mangert WF, et al.^3^ in 1969. Since then, numerous articles have appeared about its usefulness in pelvic hemorrhage, whether postpartum or related to gynecological surgery.

Ligation of IIA can be a lifesaving procedure for patients with massive hemorrhage from pelvic viscera. This is especially true in obstetrics and gynecology where hemorrhage remains a major cause of mortality and significant morbidity. Both short-term and long-term effects of IIA ligation are generally salutary. Often in severe obstetric hemorrhage, it is a life-saving procedure when other conservative measures fail. But waiting too long to perform it is its biggest pitfall.^4^

## ANATOMICAL CONSIDERATIONS

At the level of the fourth and fifth lumbar vertebra, the abdominal aorta bifurcates into left and right common iliac arteries and each further bifurcates into two main branches - the external iliac artery (which becomes the femoral artery at the inguinal ligament) and the internal iliac artery (IIA) which descends into the true pelvis. The IIA on either side arises at the level of the lumbosacral intervertebral disc and in front of sacroiliac joints. Internal iliac or hypogastric artery courses infero-medially over the pelvic brimand runs down to the pelvic cavity. At the upper margin of the greater sciatic foramen about 3.5-5.0 cm from its origin, it divides into an anterior division that continues in line with the parent vessel towards the ischial spine and the posterior division which passes backward towards the foramen. It is the main vascular supply to the pelvic viscera, gluteal muscles, and perineum. Anterior division (visceral supply) runs anteriorly along the lateral pelvic wall and supplies most of the pelvic viscera. The posterior division (parietal supply) runs posteriorly to the pelvic wall and gluteal region.^5^

**Figure 1:**
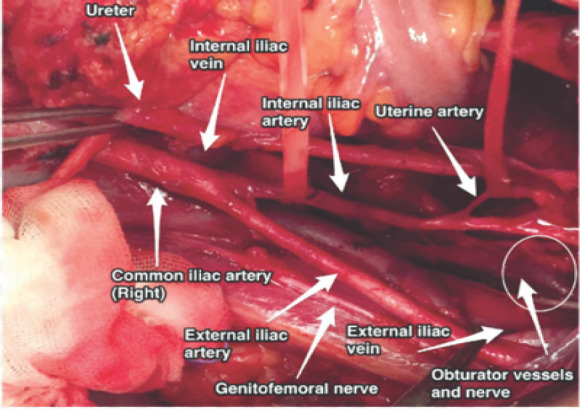
Anatomical relation of right iliac arteries in relation to the right pelvic sidewall, psoas muscle, right ureter, and nerves.

**Figure 2:**
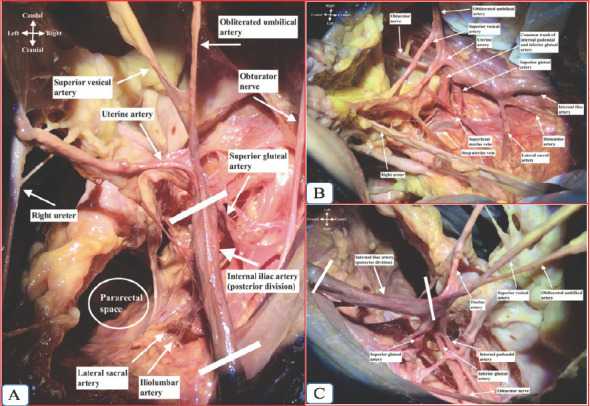
Right internal iliac artery dissection and its branches. A. Superior view; B. Medial view; C. Lateral view.

**The various branches of IIA are depicted below (Table 1, Figure 1, and Figure 2).**

**Table 1:** Branches of the internal iliac artery.

Anterior Division	
Visceral branches	Parietal branches
Uterine artery	Internal pudendal artery
Middle rectal artery	Inferior gluteal artery
Vaginal artery	Obturator artery
Superior vesical artery	
Inferior vesical artery	
Posterior Division	
Visceral branches	Parietal branches
Nil	Iliolumbar artery
	Lateral sacral artery
	Superior gluteal artery

The important anatomical relations of IIA are:^6,7^

1. Anterior - ureter;
2. Posteromedial - internal iliac vein;
3. Posterolateral - external iliac vein, obturator vein, and obturator nerve;
4. Anteromedial - covered by peritoneum with the terminal end of the ileum and cecum on the right side;
5. Lateral - Psoas major muscle, internal obturator muscle.

## CLINICAL INDICATIONS OF IIA LIGATION

There are no clear-cut guidelines for prophylactic or therapeutic indications of IIA ligation(Table 2) in pelvic surgery. Good clinical judgment is essential and it should not be delayed if prophylactic ligation is deemed to be the best course. In some circumstances, total or subtotal hysterectomy alone may not be sufficient to control ongoing pelvic hemorrhage so IIA ligation should not be delayed in such life-threatening situations. IIA ligation has a proven success rate in controlling massive pelvic hemorrhage, ranging between 40% and 100%, and obstetric indications occupy the first place as the leading factor.^8^

**Table 2:** Indications of IIA ligation.^2,6^

Obstetric indications
Atonic postpartum hemorrhage
Uterine laceration or rupture
Postpartum hemorrhage secondary to abruption or placenta previa
Morbidly adherent placenta
Post-abortion bleeding
Abdominal pregnancy with pelvic implantation of the placenta
Bleeding secondary to cervical or cesarean scar pregnancy
Before hysterectomy when all conservative measures have failed
High risk for recurrent postpartum hemorrhage Jehovah’s Witnesses with high risk for postpartum hemorrhage
Traumatic postpartum hemorrhage from the genital tract like the vagina and cervix
Gynecological indications
Significant bleeding from the broad ligament
Profuse bleeding from the pelvic sidewalls
Gynecological oncological procedures like radical hysterectomy or exenteration
Large tumor at the deeper part of the pelvis
Non-gynecological indications
Profuse pelvic bleeding without an identifiable vascular bed
Profuse pelvic hemorrhage from fracture of pelvis or gunshot injury to the pelvis
During endovascular repair of aortoiliac arterial aneurysms
Supra-levator hematoma not responding to conservative management

## SURGICAL TECHNIQUES AND PHYSIOLOGY OF IIALIGATION^2,5,6^

IIA is ligated either by transperitoneal (Figure 3) - preferred or extraperitoneal approach. All pelvic surgeons should be aware of the indications, timing, and surgical techniques of IIA ligation. A midline rather than transverse skinincision is preferred with bilateral ligation over to unilateral ligation as hemostasis is more secure and there is greater confidence in the decision not to re-explore.

The surgeon should stand where it is most comfortable during the procedure and particularly helpful to deal with each IIA from the opposite side of the operating table. With the patient in Lloyd Davis position, the abdomen is opened and the viscera packed away in the usual manner. After cutting the posterior leaf of the broad ligament and pelvic peritoneum at the lateral side of the pelvic wall, the retroperitoneal area is visualized. The incision is extended to the paracolic area parallel to the infundibulopelvic (IP) ligament. The posterior leaf of the broad ligament containing the ureter and IP ligament is pulled medially. The common iliac artery is seen over the sacroiliac joint (the ureter crosses over it).

The internal iliac artery is identified over the pelvic brim after the adipose and lymphatic tissue is retracted over the vessels. When the adipose and lymphatic tissue is dissected bluntly over the IIA along its direction (but not across to avoid unnecessary trauma), the posterior division is identified at the first 3-4 cm. After this part, the anterior division is seen and ligation is performed at this point. If the artery is difficult to visualize, it is better to feel for a pulsation (difficult to palpate if a patient is hypotensive).

Meticulous dissection is required to separate the IIA from the veinbefore ligation if they are adherent. It is advised to try separating the artery and the vein by opening the tips of the blunt right-angled forcepsslowly until a path has been found between them. A right angle, blunt-ended forceps is passed laterally to medial between the artery and the vein upon which is threaded a non-absorbable suture such as 1-0 silk or vicryl around the anterior division of the IIA. The choice of material for ligation depends on the preference of the surgeon. Two ties should be placed gently but firmly in continuity approximately 0.5 cm apart.

Burchell^2^ has described the mechanism responsible for controlling pelvic hemorrhage following ligation of IIA. The IIA ligation significantly reduced the pulse pressure and the pelvic arterial system is converted into a venous system with sluggish blood flow. With bilateral ligation, the fall in pulse pressure was 85% while it was 77% on the same side and 14% on the opposite side following unilateral ligation. Similarly, the fall in mean arterial pressure was 24% with bilateral ligation but it was 22% on the same side and 10% on the opposite side with unilateral ligation. The rate of blood flow dropped to about 48% on the same side following ligation.

The mean pressure remained more than the venous pressure, but the ‘trip-hammer effect’ of arterial pulsation was abolished. This reduction in pulse pressure permits effective thrombosis to take place so that small vessels stop bleeding. After ligation, clot once formed have a strong chance of remaining in place unless it was mechanically wiped away. Decreased pulse pressure, the basic hemostatic effect of IIA ligation, was due to the small caliber of the pelvic anastomoses involved in the collateral blood supply. Burchell^2^ observed that there was free blood flow from a severed uterine artery even after bilateral IIA Ligation. After ligation of IIA, ample collateral circulation in the parts supplied by the IIA develops because of major pelvic anastomoses (Table 3).Collateral circulation is functional as early as 45-60 minutes after ligation. No tissue necrosis develops after IIA ligation because the pelvic blood supply is unbelievably abundant. The diameter of collateral anastomoses does not increase with time with no compensatory growth because the pelvic blood supply is so enormous.

**Table 3:** Major pelvic anastomoses after IIA ligation.^6^

Branches of IIA	Anastomotic vessels
1. Uterine artery	1. Right and left ovarian arteries (direct branches of the aorta)
2. Inferior and middle hemorrhoidal arteries	2. Superior hemorrhoidal artery (branch of inferior mesenteric artery)
3. Pubic branches of the obturator artery	3. Inferior epigastric artery (branch of external iliac artery)
4. Inferior gluteal artery	4. Circumflex and perforating branches of the deep femoral artery
5. Superior gluteal artery	5. Lateral sacral artery (posterior branches)
6. Iliolumbar artery	6. Lumbar artery (from the aorta)
7. Lateral sacral artery	7. Middle sacralartery
8. Vesical artery	8. Branches of the uterine and vaginal arteries

## OPERATIVE COMPLICATIONS DURING IIA LIGATION^6^

The complications which could be encountered during the ligation of IIA are accidental injury to the internal iliac artery and/or vein, ligation or laceration of the external iliac artery, ligation or laceration of the ureter, incorporation of the anterior division of the sciatic nerve, disseminated intravascular coagulation and rare injury to the rectum. It is essential to open the pelvic peritoneum over the pelvic vessels carefully, dissect the retroperitoneal - pelvic spaces to identify the internal iliac artery clearly, and reflect the peritoneum containing the ureter medially.

Ligation of the external iliac artery produces an acutely ischemic leg which classically presents with the absence of distal pulses and whiteness or pallor of the foot. It is essential to check for a pulse in the external iliac artery above the inguinal ligament, beyond the area of ligation; the femoral pulse in the groin; or the vascular flow on doppler at the ankle, if there is a high suspicion that the main artery to the lower limb is ligated. If the wrong artery has been ligated, the ligature should be removed. If this fails to restore a good pulse, a vascular surgeon should be called to repair the vessel.

Similarly, accidental damage to either or both ureters may occur in life-threatening surgery to control massive pelvic hemorrhage. The ligature is more probable than transection. Prompt diagnosis and remedial surgery by a urological consultation are essential. Damage to the ureter should be avoided by careful dissection and visualization of pelvic structures before ligating IIA.

Damage to the iliac vein or one of its major tributaries results in brisk hemorrhage. If sudden venous bleeding occurs, the first step is to apply firm pressure to the area. Adequate suction should be prepared. Swabs on sponge-holding forceps can then be applied on both sides of the site of damage to compress the veins and allow the defect to be visualized. After the defect is seen, its edges can be held together using a traumatic forceps to repair with a non-absorbable vascular suture on a round-bodied needle.

Other uncommon complications include ischemic necrosis of the gluteal region, foot drop, paresthesia of the gluteal region, lower limb amputation, or bladder atony.

The major pitfall associated with ligation of the IIA is a delay. With the state of hemorrhagic shock in irreversible decompensation, this operation will not overcome it. The inadequate transfusion is another pitfall in the therapy of patients with severe hemorrhage. Blood loss is often seriously underestimated. Failure to remember that the vaginal artery is a separate branch of the IIA rather than a branch of the uterine artery may lead the surgeon into the pitfall of an unnecessary hysterectomy for control of bleeding.

## FERTILITY ISSUES^9^

Fertility, menstrual, and reproductive capacity is not affected due to good collateral circulation provided that the patient has a normal uterus. There have been reports of normal pregnancy and term delivery occurring after bilateral IIA ligation. These collaterals undergo hypertrophy during a subsequent pregnancy. This was confirmed by the demonstration of normal uterine and fetal circulation monitored with Color Doppler in pregnancy. Intrauterine growth restriction has not been reported suggesting adequate blood supply after ligation.

## WAY FORWARD

Pelvic hemorrhage is a major cause of maternal morbidity and mortality in developing countries. Internal iliac ligation is a valuable surgical procedure and should be considered with no delay where conservation of the uterus is desired in young women with intractable pelvic hemorrhage giving the chance of future fertility. It is also rewarding to control hemorrhage after obstetric hysterectomy and hemorrhage after gynecological surgeries. Every pelvic surgeon needs to learn life-saving procedures like internal iliac artery ligation with an understanding of retroperitoneal anatomy and regional variations of the internal iliac artery to reduce the risk of operative complications.

Pelvic arterial embolization nowadays is an upcoming alternative life-saving procedure to internal iliac artery ligation and hysterectomy where arteriographic facilities are available.
